# Fluoride ion adsorption onto palm stone: Optimization through response surface methodology, isotherm, and adsorbent characteristics data

**DOI:** 10.1016/j.dib.2017.04.030

**Published:** 2017-04-29

**Authors:** Masoumeh Ravanipour, Raheleh Kafaei, Mozhgan Keshtkar, Soghra Tajalli, Narjes Mirzaei, Bahman Ramavandi

**Affiliations:** Department of Environmental Health Engineering, Faculty of Health and Nutrition, Bushehr University of Medical Sciences, Bushehr, Iran

**Keywords:** Palm stone, Response surface methodology, Fluoride ion, Aqueous solution, Optimization

## Abstract

In some part of the world, groundwater source can become unsafe for drinking due to the high concentration of fluoride ions [1]. The low cost and facile-produced adsorbent like palm stone could effectively removed fluoride ions through adsorption process. In this dataset, the influence of fluoride ion concentration, solution pH, adsorbent dosage, and contact time on fluoride ion adsorption by palm stones was tested by central composite design (CCD) under response surface methodology (RSM). The data stone carbonized adsorbent was prepared by a simple and facile method at relatively low temperature of 250 °C during 3 h. The adsorbent had the main functional groups of O–H, –OH, Si–H, C=O, N=O, C–C, C–OR, C–H, and C–Br on its surface. At the optimized conditions obtained by RSM, about 84.78% of fluoride ion was removed using the adsorbent. The Langmuir isotherm was suitable for correlation of equilibrium data (maximum adsorption capacity= 3.95 mg/g). Overall, the data offer a facile adsorbent to water and wastewater works which face to high level of fluoride water/ wastewater content.

**Specifications Table**

**Table:** 

Subject area	*Chemical engineering*
More specific subject area	*Environmental engineering*
Type of data	*Table, image, figure*
How data was acquired	*All adsorption tests were done in batch mode. Five level of each parameter was evaluated using RSM.* *The concentrations of fluoride in the samples were measured using a UV-visible spectrophotometer (HACH, USA, model CAM Spec M501) with a standard SPADNS reagent at 570 nm.* *A digital pH meter (Metrohm) was used for solution pH analyzing.* *The characteristics of the adsorbent were analyzed using FTIR (VERTEX 70/70 v), SEM (JSM- 5510, Jeol Ltd., Tokyo, Japan), XRD (Philips X’Pert, Netherlands) and pHzpc.*
Data format	*Analyzed*
Experimental factors	*Measuring of F concentrations under various levels of initial F concentration, solution pH, adsorbent dosage, and contact time to obtain optimal F removal from aqueous solution using an adsorbent provided from palm stone.*
Experimental features	Optimization of F adsorption onto palm stone adsorbent using RSM
Data source location	*Bushehr University of Medical Sciences, Bushehr, Iran, GPS: 28.9667°N, 50.8333°E*
Data accessibility	*Data represented with the article*

**Value of the data**

• This data offer a simple method for preparation of adsorbent from palm stones.
• This data article presents a user friendly- statistical method (RSM) to optimize fluoride ion removal from aqueous solution using adsorption process.
• The dataset will be useful for fluoride ion removal from waters and wastewaters.

## 1 Data

**Fig. 1:**
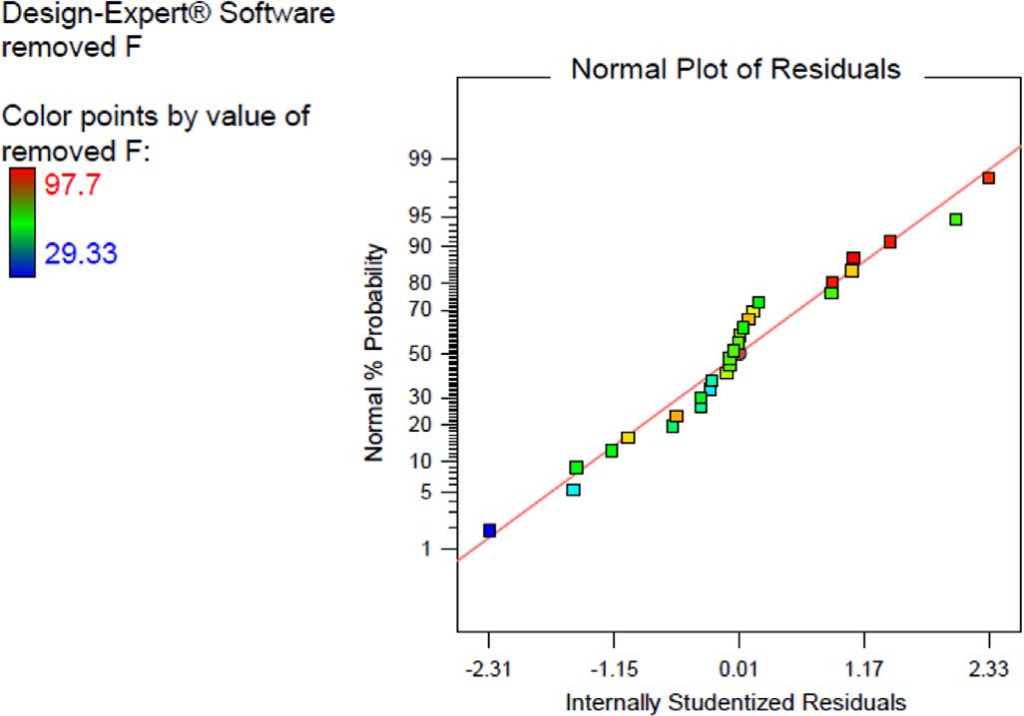
Normal probability plot and residual versus fit plot for fluoride removal efficiency.

**Fig. 2:**
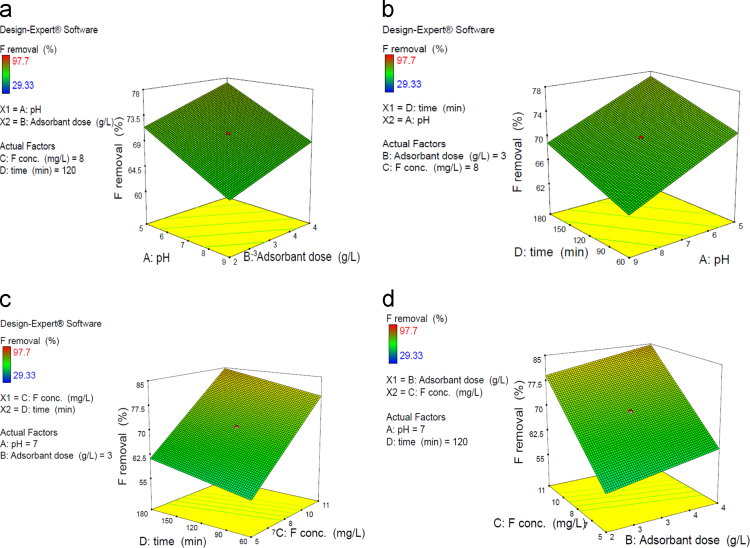
Central composite design 3-D surface plots showing effect of (a) p1H and adsorbent dosage, (b) contact time and pH, (c) contact time and F concentration, (d) F concentration and adsorbent dosage on fluoride removal efficiency with the adsorbent.

**Fig. 3:**
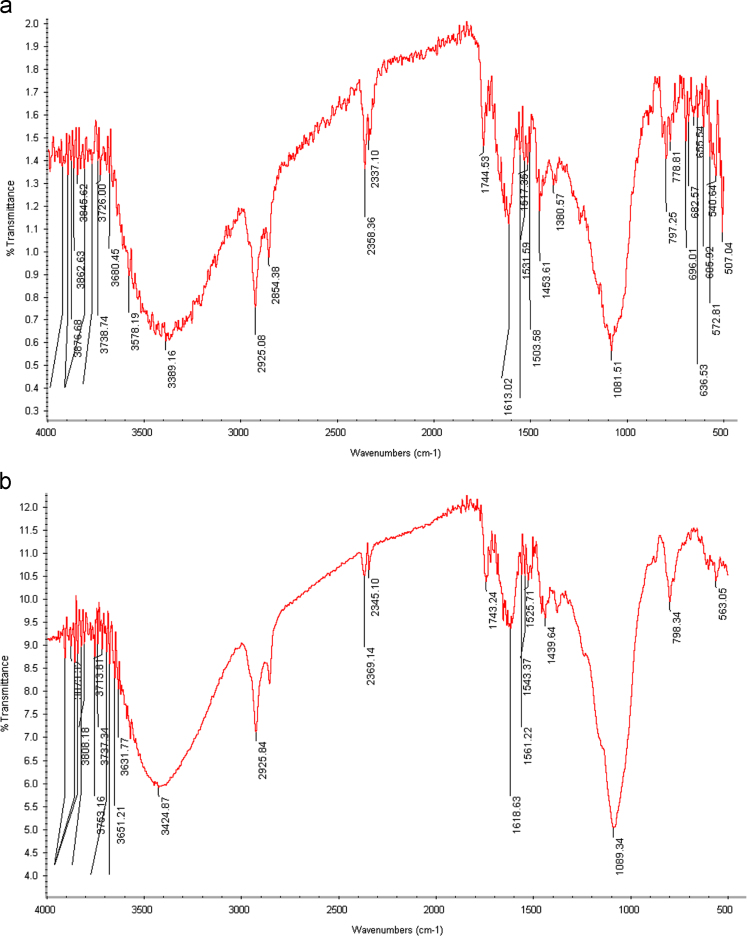
The FTIR spectra for (a) fresh and (b) used adsorbent in the F adsorption.

**Fig. 4:**
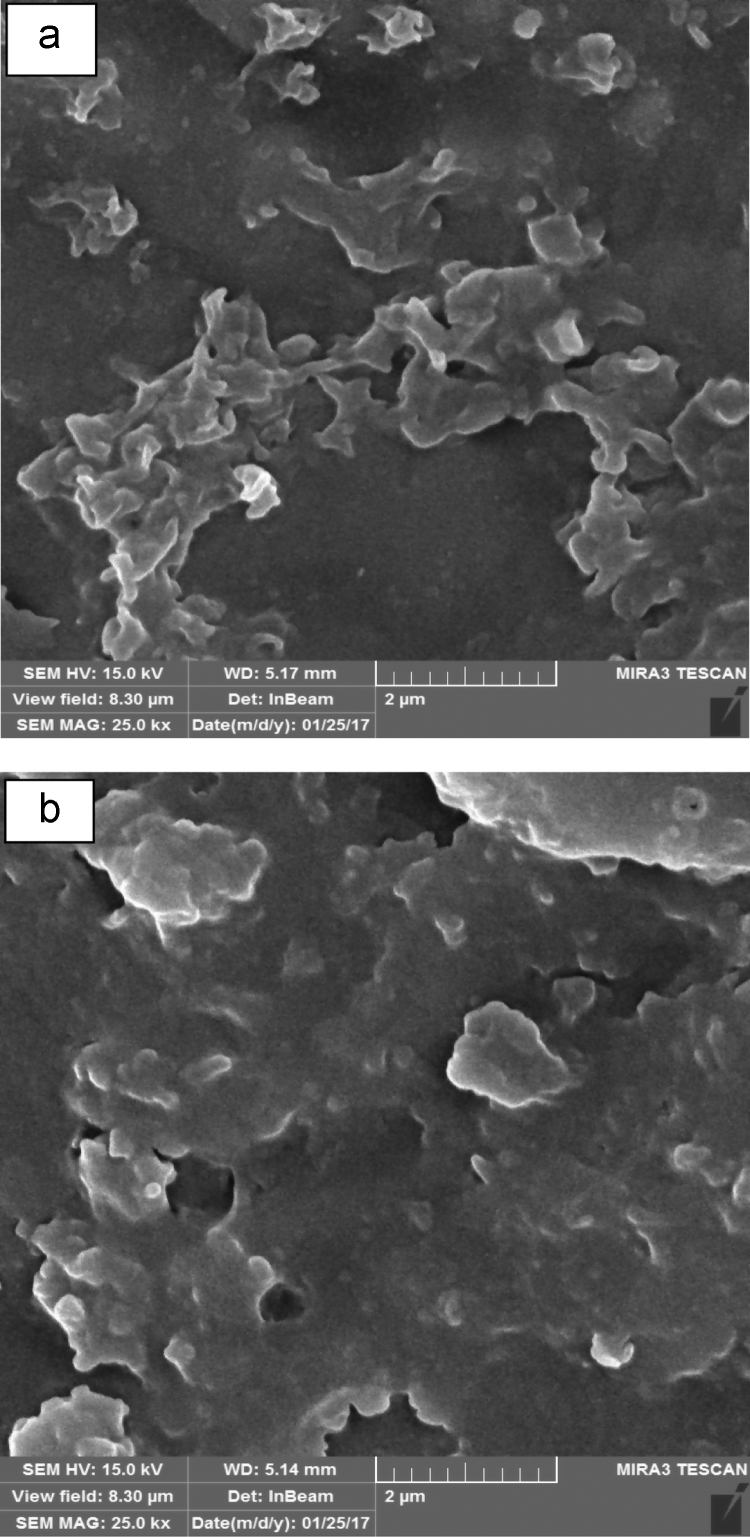
The SEM images of (a) fresh and (b) used adsorbent in the F adsorption.

**Fig. 5:**
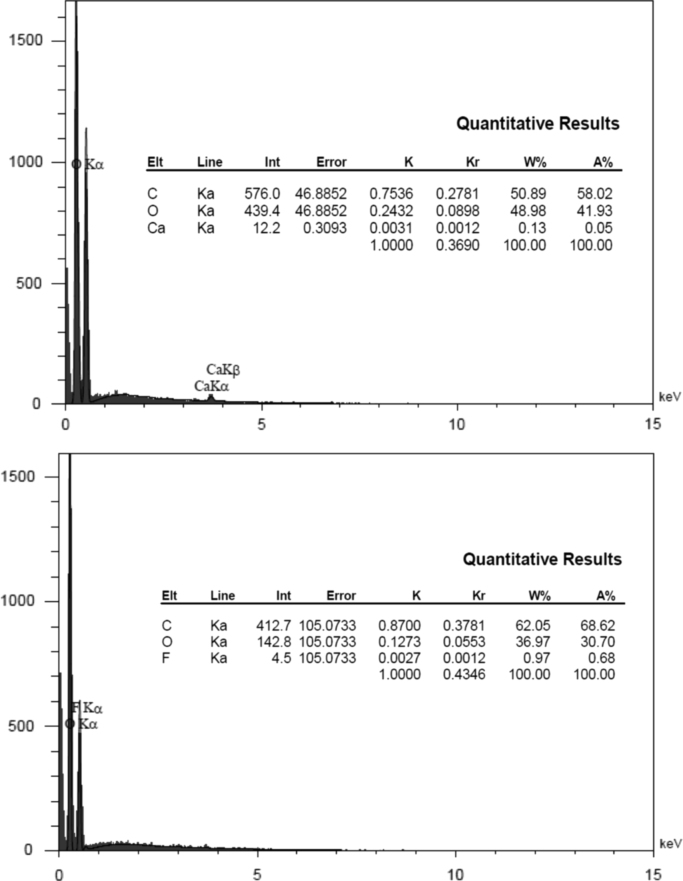
The XRD images of (a) fresh and (b) used adsorbent in the F adsorption.

**Fig. 6:**
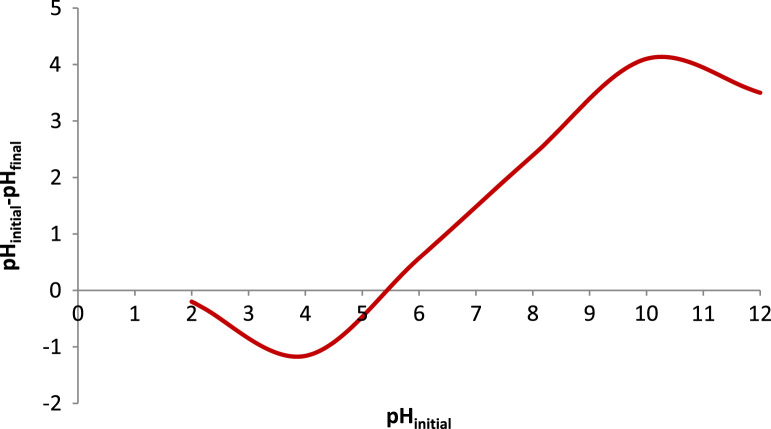
Variation of pH (pH_initial_ – pH_final_) versus initial pH for determining the adsorbent pH_zpc_.

Table 1 in this data article contains data for independent variables and their coded levels to central composite design. Normal probability plot and residual versus fit plot for fluoride adsorption efficiency are depicted in Fig. 1. Central composite design 3-D surface plots which showing effect of various parameters on fluoride removal efficiency with the adsorbent are presented in Fig. 2. The data for model summary statistics and ANOVA for central composite design are listed in Table 2, Table 3. The FTIR spectra for fresh and used adsorbent in the F adsorption are also depicted in Fig. 3. The surface morphology (SEM) of the adsorbent was presented in Fig. 4. The XRD analysis was used to explore fresh and used adsorbent structure; the results of this analysis are shown in Fig. 5. The pHzpc factor which is important for explanation the pH effect on the removal of pollutant [1], [2] is seen in Fig. 6. Table 4 shows isotherm models data used in this article.

## 2 Experimental design, materials and methods

### 2.1 Adsorbent preparation

The palm stones used in this study were prepared from a local agricultural palm field in Bushehr province, Iran. All palm trees were belonged to *Phoenix dactylifera* species. After separating the stones from dates, they were washed three times by distilled water and dried at 105 °C for 3 h. Dried stones were milled, grinded and sieved in the size ranged from 1 to 2 mm [5], [6]. These sieved stones were carbonized in the oven in 250 °C for 3 h, and then cooled and stored in a plastic bag under the desiccators and used for the adsorption tests. Thus, this adsorbent could easily provide with a simple method, however, the production of palm date wastes (the base material for the adsorbent) is time depended.

### 2.2 Adsorption tests

Response surface methodology may be summarized as a collection of statistical tools and techniques for constructing and exploring an approximate functional relationship between a response variable and a set of design variables. This experimental method has high finding efficiency for the operating conditions at least of cost, while give good knowledge about variable interaction [7], [8], [9]. The effect of four parameters was evaluated by using Central Composite Design (CCD). Each factor in the experimental design was studied at five different levels as shown in Table 1. Experiments were planned in 2^4^ trials plus 4 centre points, and 8 axial points. Thus, twenty eight experiments were conducted at the room temperature (25±1 °C). The Design Expert software (version 7.0, Stat-Ease) was used for data analyzing.

The working fluoride solutions were prepared via diluting the stock solution 1000 mg/L of sodium fluoride (Germany, Merck Co.), in Erlenmeyer flasks to obtain concentrations (2, 5, 8, 11, and 14 mg/L). The flasks were agitated at 120 rpm in different contact times, adsorbent dosages and pH in order to reach the adsorption equilibrium conditions. After the completion the contact time, the solution was filtrated using the Whatman filter paper (pore size 0.45 μm). The filtrate solution was analyzed for residual fluoride concentration. The solution pH was adjusted using 0.1 N HCl or NaOH solutions. Blank solution was undertaken to evaluate self aggregation and or settling of adsorbate during the experiments.

The experiments were carried out in batch mode. The following equation was used for calculation of the adsorption percentage [10], [11], [12]:

(1) $$\mathrm{Adsorption percentage}=\frac{(C_{i}-C)}{C_{i}}\times 100$$

Where: *C*_*i*_ and *C* are the initial and final fluoride concentration (mg/L), respectively.

We noted that the optimized F removal value by the Design Expert software and confirmation test were achieved 84.78 and 81.55%, respectively. Further, by using regression analysis on the data, the final equation in terms of actual parameters was obtained:

(2) Fremoval(%)=39.23413-2.065×pH+2.789167×Adsorbentdose+3.915556×Fconc.+0.047375×Time

### 2.3 Measurements

The residual fluoride concentration was measured by using a UV–Vis spectrophotometer (HACH, USA, model CAM Spec M501) with a standard SPADNS reagent (Germany, Merck Co.) at 570 nm according to the Standard Methods of Examination of Water and Wastewater [13]. The solution pH was also adjusted using 0.1 N HCl or NaOH solutions and measured by a digital pH meter (827 pH labs Metrohm AG, Herisau, Switzerland). Fourier transform infrared spectroscopy (FTIR) spectrum of the fresh and used adsorbent samples was obtained using VERTEX 70/70 v FT-IR spectrometers. The FTIR data showed that the functional groups of O–H, –OH, Si–H, C=O, N=O, C–C, C–OR, C-H, and C-Br are probably played role in the adsorption process [14], [15], [16]. The morphology of the adsorbent particles was performed by scanning electron microscopy (JSM- 5510, Jeol Ltd., Tokyo, Japan) under an acceleration voltage of 30 kV. The XRD pattern of the fresh and used adsorbent was recorded by an automated X-ray diffracto-meter (Philips X’Pert, Netherlands) at the condition explained in the literature [17], [18]. The pHzpc factor was obtained using method specified by published studies [19], [20], [21], briefly; in the batch equilibrium method we used the ratio of 1:250 adsorbent to distilled water in 0.01 M NaCl solution, as an inert electrolyte.

## Figures and Tables

**Table 1:** Independent variable and their coded levels to central composite design.

Code	Variable	-α	-1	0	1	+α
A	pH	3	5	7	9	11
B	Adsorbent dose (g/L)	1	2	3	4	5
C	Fluoride conc. (mg/L)	2	5	8	11	14
D	Time (min)	0	60	120	180	240

**Table 2:** Analysis of variance (ANOVA) data for central composite design.

Source	Sum of Squares	df	Mean Square	F Value	p-value	
					Prob >F	
Model	4101.60	4	1025.40	7.029	0.0008	Significant
A-pH	409.36	1	409.36	2.806	0.1074	
B-Adsorbent dose	186.70	1	186.70	1.279	0.2696	
C-F conc.	3311.62	1	3311.62	22.702	<0.0001	
D-Time	193.91	1	193.91	1.329	0.2608	
Residual	3354.99	23	145.86			
Lack of Fit	3354.44	20	167.72			
Pure Error	0.54	3	0.1820			
Cor Total	7456.60	27				

**Table 3:** Model summary statistics.

Source	Std. Dev.	R-Squared	Adjusted	Predicted	PRESS	
			R-Squared	R-Squared		
Linear	12.07	0.55	0.471	0.292	5272.68	Suggested
2FI	11.26	0.71	0.540	-0.058	7894.64	
Quadratic	12.28	0.74	0.453	-0.515	11,298.12	
Cubic	5.38	0.98	0.895	-1.789	20,796.77	Aliased

**Table 4:** Isotherm models used in this dataset [3], [4].

Langmuir				Freundlich			
Equation	Q_m_	R^2^	K_L_	Equation	K_f_	n	R^2^
Y=0.9814 X+0.2533	3.95	0.9309	0.258	Y=0.5461X - 0.099	0.796	1.831	0.7936

Langmuir ($q_{e}=\frac{Q_{m}K_{L}C_{e}}{1+K_{L}C_{e}}$):

q_e_= the amount of adsorbed fluoride per gram of adsorbent at equilibrium (mg/g), Ce= equilibrium fluoride concentration (mg/L), *Q*_*m*_ = maximum adsorption capacity (mg/g), *K*_*L*_= Langmuir constant (L/mg)

Freu*n*dlich ($q_{e}=K_{f}{C_{e}}^{1/n}$):

*K*_*f*_ = Freundlich constant, *n* = Freundlich constant (mg/g(L/mg)^1/n^)

## References

[1] Papari F., Rouhi Najafabadi P., Ramavandi B. (2017). Fluoride ion removal from aqueous solution, groundwater, and seawater by granular and powdered Conocarpus erectus biochar. Desal. Water Treat..

[2] Sharifpour E., Haddadi H., Ghaedi M. (2017). Optimization of simultaneous ultrasound assisted toxic dyes adsorption conditions from single and multi-components using central composite design: application of derivative spectrophotometry and evaluation of the kinetics and isotherms. Ultrason. Sonochem..

[3] M. Ahmadi, M. Foladivand, N. Jafarzadeh, B. Ramavandi, S. Jorfi, B. Kakavandi, Synthesis of chitosan zero-valent iron nanoparticles-supported for cadmium removal: characterization, optimization and modeling approach, J. Water Supply Res. T., 66 (2), pp. 116–130.

[4] F.S. Sarvestani, H. Esmaeili, B. Ramavandi, Modification of Sargassum angustifolium by molybdate during a facile cultivation for high-rate phosphate removal from wastewater: structural characterization and adsorptive behavior, 3 Biotech, 6 (2), p. 251. 10.1007/s13205-016-0570-z PMC5120031 28330323

[5] Khademi Z., Ramavandi B., Ghaneian M.T. (2015). The behaviors and characteristics of a mesoporous activated carbon prepared from Tamarix hispida for Zn (II) adsorption from wastewater. J. Environ. Chem. Eng..

[6] Ramavandi B., Asgari G., Faradmal J., Sahebi S., Roshani B. (2014). Abatement of Cr (VI) from wastewater using a new adsorbent, cantaloupe peel: taguchi L_16_ orthogonal array optimization. Korean J. Chem. Eng..

[7] Askari H., Ghaedi M., Dashtian K., Azghandi M.H.A. (2017). Rapid and high-capacity ultrasonic assisted adsorption of ternary toxic anionic dyes onto MOF-5-activated carbon: artificial neural networks, partial least squares, desirability function and isotherm and kinetic study. Ultrason. Sonochem..

[8] Dastkhoon M., Ghaedi M., Asfaram A., Arabi M., Ostovan A., Goudarzi A. (2017). Cu@ SnS/SnO_2_ nanoparticles as novel sorbent for dispersive micro solid phase extraction of atorvastatin in human plasma and urine samples by high-performance liquid chromatography with UV detection: application of central composite design (CCD). Ultrason. Sonochem..

[9] Dastkhoon M., Ghaedi M., Asfaram A., Goudarzi A., Mohammadi S.M., Wang S. (2017). Improved adsorption performance of nanostructured composite by ultrasonic wave: optimization through response surface methodology, isotherm and kinetic studies. Ultrason. Sonochem..

[10] Asgari G., Ramavandi B., Sahebi S. (2014). Removal of a cationic dye from wastewater during purification by Phoenix dactylifera. Desal. Water Treat..

[11] Rahbar A., Farjadfard S., Leili M., Kafaei R., Haghshenas V., Ramavandi B. (2016). Experimental data of biomaterial derived from Malva sylvestris and charcoal tablet powder for Hg^2+^ removal from aqueous solutions. Data Brief.

[12] Ghaedi M., Khafri H.Z., Asfaram A., Goudarzi A. (2016). Response surface methodology approach for optimization of adsorption of Janus Green B from aqueous solution onto ZnO/Zn (OH) 2-NP-AC: kinetic and isotherm study. Spectrochim. Acta Mol. Biomol. Spectrosc..

[13] McGraw Hill A. (2005). Standard Methods of Examination of Water and Waste Water.

[14] Ardekani P.S., Karimi H., Ghaedi M., Asfaram A., Purkait M.K. (2017). Ultrasonic assisted removal of methylene blue on ultrasonically synthesized zinc hydroxide nanoparticles on activated carbon prepared from wood of cherry tree: experimental design methodology and artificial neural network. J. Mol. Liq..

[15] Bagheri A.R., Ghaedi M., Asfaram A., Jannesar R., Goudarzi A. (2017). Design and construction of nanoscale material for ultrasonic assisted adsorption of dyes: application of derivative spectrophotometry and experimental design methodology. Ultrason. Sonochem..

[16] Daneshyar A., Ghaedi M., Sabzehmeidani M. (2017). H_2_S adsorption onto Cu-Zn–Ni nanoparticles loaded activated carbon and Ni-Co nanoparticles loaded γ-Al_2_O_3_: optimization and adsorption isotherms. J. Colloid Interface Sci..

[17] Dashamiri S., Ghaedi M., Dashtian K., Rahimi M.R., Goudarzi A., Jannesar R. (2016). Ultrasonic enhancement of the simultaneous removal of quaternary toxic organic dyes by CuO nanoparticles loaded on activated carbon: central composite design, kinetic and isotherm study. Ultrason. Sonochem..

[18] Azad F.N., Ghaedi M., Dashtian K., Hajati S., Pezeshkpour V. (2016). Ultrasonically assisted hydrothermal synthesis of activated carbon–HKUST-1-MOF hybrid for efficient simultaneous ultrasound-assisted removal of ternary organic dyes and antibacterial investigation: Taguchi optimization. Ultrason. Sonochem..

[19] Ahmadi M., Kouhgardi E., Ramavandi B. (2016). Physico-chemical study of dew melon peel biochar for chromium attenuation from simulated and actual wastewaters. Korean J. Chem. Eng..

[20] Asgari G., Mohammadi A.S., Mortazavi S.B., Ramavandi B. (2013). Investigation on the pyrolysis of cow bone as a catalyst for ozone aqueous decomposition: kinetic approach. J. Anal. Appl. Pyrol..

[21] Jamshidi M., Ghaedi M., Dashtian K., Hajati S., Bazrafshan A. (2016). Sonochemical assisted hydrothermal synthesis of ZnO: cr nanoparticles loaded activated carbon for simultaneous ultrasound-assisted adsorption of ternary toxic organic dye: derivative spectrophotometric, optimization, kinetic and isotherm study. Ultrason. Sonochem..

